# Simply red? The effects of distinct colours and sustainable production methods on the consumers’ preferences for healthier sweet peppers

**DOI:** 10.1016/j.heliyon.2024.e28661

**Published:** 2024-04-12

**Authors:** Giuseppe Di Vita, Raffaele Zanchini, Daniela Spina, Antonella Vastola, Mario D'Amico, Francesco Caracciolo

**Affiliations:** a Department of Agriculture, Food and Environment (Di3A), University of Catania, Catania, Italy; b Department of Agricultural, Forest and Food Science (DISAFA), University of Turin, Grugliasco, Italy; c Department of Agriculture, University of Naples Federico II, Portici, Italy

**Keywords:** Antioxidant, Consumers' preferences, Healthy choices, Wellbeing, Organic products, Integrated pest management

## Abstract

The purpose of this paper is to thoroughly assess the value of colours in consumers' preferences for sweet peppers, and the association with more sustainable methods of production in the consumers' minds. Furthermore, this study provides novel insights into the influence of colours on the willingness to pay (WTP) for vegetables. It explores the interplay between colours, food attributes, and socio-demographic characteristics among consumers, marking the first attempt to examine this relationship comprehensively. A discrete choice experiment, based on consumers' preferences for sweet pepper attributes (price, colour, and production method), was implemented and a Seemingly Unrelated Regression model was then applied to evaluate the willingness to pay for different colours. The study has revealed that different colours provide consumers with different utilities and WTP in the choices of potential healthier foods. In addition, gender, age, education and number of family components play a role in affecting consumers' WTP of food linked to colour. Finally, it was found that consumers’ knowledge for certain pepper colours with different antioxidant contents is interrelated. This study introduces several novelties, in particular a positive correlation between interest in antioxidants and colours was found, suggesting that interest in healthy food properties might move consumers towards a specific sensory choice.

## 1 Introduction

Wellbeing is perceived as a multidimensional construct within the food consumption context [1,2], and it depends on several factors related to beliefs, physical health, psychological traits and emotional state [3,4], but it cannot be separated from the sensory characteristics of food products.

The visual appearance of food, as based on a different range of colours, appears to be one of the most relevant sensory attributes. Indeed, the visual perception of consumers is a crucial aspect in their choices of unprocessed food, and in particular of fruit and vegetables [5]. In this regard, some studies have highlighted an association between colour and a corresponding healthiness of food [6]. The colour in fact denotes higher or lower concentrations of antioxidants and total carotenoids, which increase as a result of the adopted agronomic practices, fruit ripening and according to the type of cultivar [7,8].

Since the colour of vegetables is determined by different concentrations of colouring substances, such as carotenoids and anthocyanins [9], it could be interesting to understand whether consumers implicitly assign a particular value to the colour aspect. It, being linked to different content of antioxidants, not only influences the sensory perception but also it can consequently determine more or less healthful choices [10].

For these reasons, and because of the colour variability of different cultivars, sweet peppers were chosen as a case study to deepen the understanding of a certain relationship between colour and health resulting from antioxidant content. Peppers, thanks to their high phenolic content, which varies according to the season and hence to their colour, have certain functional properties and antioxidant properties, and as such they are suitable for helping to overcome certain health-related issues. In fact, they contain several types of polyphenols, flavonoids, and capsaicinoids [11] and have a strong antioxidant activity [12]. Empirical evidence has shown that polyphenols enhance the endogenous antioxidant defence system. In addition, they also have an anti-carcinogenic and antimutagenic effect, as they inhibit DNA oxidation, slow down the development of malignant tumours and help to prevent cardiovascular disease and neurological disorders [[13], [14], [15]].

Moreover, although sweet peppers are largely consumed in Mediterranean countries as a fresh product, or as an ingredient in cooked or processed food [11,16], there is a certain paucity of studies regarding the association between colour and the potential choices of consumers. This relation can be investigated using consumers' knowledge about antioxidant properties of peppers to understand if a correlation with choices exists. To the best of the authors' knowledge, no study has analysed the different levels of knowledge in relation of healthfulness of food choices linked to different antioxidant properties and conveyed by a different chromatism. Therefore, this study represents first exploratory research in investigating the impact of colour on consumers’ willingness to pay for vegetables, contributing to the existing body of literature on colour preference in food packaging [17], salmon [18], and eggs [19].

In the light of these premises, the aim of the present paper is to evaluate in detail the value of colours in consumers’ preferences. Through a choice method experiment, this study aims to classify the hierarchical utility of sweet pepper consumers according to different attributes, such as colour and sustainable method of production. It also aims to verify whether awareness of healthy properties of peppers in term of antioxidant activity is interrelated with choices of different colours.

The remainder of paper consists of five sections. The first section presents a literature framework on the relationship between healthiness and sensory attributes in consumers' food choices, to present the objectives and research questions of this work. The second section provides information on how data was collected and processed. A discrete choice experiment was implemented, and a Seemingly Unrelated Regression model (SUR) was then applied to evaluate drivers of willingness to pay for different colours. The third section presents the main outcomes of the study, as derived from the double econometric approach. The fourth section discusses the results and provides additional insights and incremental knowledge on the existing literature pertaining to the role of colour in food consumers’ health choices. The last section concludes the paper by reporting the main findings and provides immediate takeaways for academicians and practitioners.

## 2 Research background, objective of the study and research questions

### 2.1 Visual perception, colour and healthiness

Recently obtained evidence has shown that colour seems to affect the behaviour of consumers, in terms of both healthiness and eating habits. It has been widely demonstrated that colour can promote healthier choices among consumers as, for example, in the case of colour coded food labels [20].

Other studies have primarily focused on evaluating the impact of package colours. A recent investigation examined the healthiness and sweetness of various food products with different coloured packaging [21]. The findings suggested that red packages are perceived as less healthy due to their association with sweetness, while blue and green are more directly linked to the perception of health. Another study highlighted that more subdued-coloured packages are associated with the idea of healthiness [22]. Additionally, some authors noted that light colour significantly influences consumers' acceptability and willingness to eat bell peppers, especially when presented under yellow or white light [23].

The colour of the exocarp of fresh fruit and vegetables is considered a synthetic index of food quality, fruit ripening and defects. It also indicates the antioxidant ability of a product [5]. The antioxidant properties are higher for magenta, blue and red-coloured fruit, due to their richness in anthocyanins. Conversely, green and white fruit and vegetables, as well as those with a high percentage of chlorophyll, have a lower antioxidant content [5].

Matsufuji et al. [24], who analysed five different coloured sweet peppers, found different antioxidant properties in the following decreasing order: red, orange, yellow, green and white. The red coloured sweet peppers had the highest total carotenoid content, while orange-coloured sweet peppers were the highest in a-tocopherol [24]. As a result, most authors agree that red sweet peppers have higher levels of carotenoids, a-tocopherol, sugars, and organic acids, as well as a stronger antioxidant activity than other cultivars, such as orange, yellow, green and white peppers [9]. Nevertheless, a previous study on the consumers of sweet peppers evidenced that green is usually the most favoured colour, and that it is preferred over the other colours, that is, orange, red, yellow, and brown [25].

To summarise, a certain relationship exists between the *subjective perception of healthy* food, due to its beneficial substance content, and its sensory attributes. In fact, **e**mpirical analyses and practical tasting assays have demonstrated that the quality of vegetables can be experienced through organoleptic attributes, and some of these analyses and assays have matched the sensory attributes of food with subjective perception of healthiness [26,27]. Consequently, colour can be used as a health cue if placed in connection with the related antioxidant properties.

Regarding the role of socio-demographic characteristics, it was observed that household size and income positively correlate with different varieties of pepper. Conversely the consumers’ willingness to pay for pepper varieties is mainly influenced by low price, taste, and thickness [28].

In light of these considerations, the current paper aims to tackle these issues. Specifically, it endeavors to discern:

H1 whether different colours provide consumers with varied utilities in their selection of healthier food, thereby resulting in distinct WTP;

H2 Whether consumers' interest in antioxidants positively influences the WTP for various colours;

H3 The influence of socio-demographic characteristics on consumers' willingness to pay for each color.

### 2.2 The role of organic and integrated pest management in the subjective perception of healthy food

The literature on the consumption of fresh vegetables produced organically is very rich in both theoretical and empirical contributions, which have highlighted that organic productions provide several perceived sustainability benefits for consumers, but also produce certain health benefits [16,29].

There is evidence that healthiness is one of the most important drivers in purchasing organic products [30]. Moreover, the absence of pesticides leads consumers to consider certain foods as healthy. Consequently, healthiness is believed as the principal motivation for buying organic products, consumers are also willing to pay an additional price for it [31]. Other studies have explored what drivers influence healthiness perception of food [27,32]. It has been found, for example, that an organic label significantly increases the perception of a food as being healthy [33].

Conversely, there are very few studies on the role of integrated pest management in consumers' behaviour. Generally, consumers evaluate the presence of an IPM label as being less important than an organic attribute [34]. Nevertheless, vegetable consumers have shown a good propensity towards this label, since the attitudes and intentions of more frequent buyers to purchase such food have been found favourable [35]. A willingness to buy has also been confirmed in another study that analysed consumers’ preferences for clementines obtained by means of an integrated pest management process [36].

Additionally, empirical evidence has shown a direct correlation between organic and integrated farming and the intensity of colours. It particular, it has been found that organic farming results in a higher intensity of colour as well as a higher content of minerals and carotenoids than integrated farming. Furthermore, peppers grown using integrated pest management practices present intermediate values of such components, as in the case of red and yellow peppers [37]. To date, no study has been carried out on the subjective perception of healthy food derived from this label.

The choice of the latter attributes is justified by the fact that they represent some of the most important food credence attributes [38,39]. They are also considered in the existing literature as the most relevant determinants of sustainable preferences for consumers, which also include health-related aspects [40]. Consequently, they have been used as a "benchmark" to measure the effect of colour on the choices of healthy food.

H4 Based on these considerations, we found it interesting to incorporate sustainable attributes associated with various production methods (organic and integrated farming) into our survey on the subjective perception of food healthiness. For the reasons stated above, it becomes compelling to examine whether each of the different production methods has a specific impact on utility and elicits a positive WTP.

### 2.3 Subjective perception of healthy food and different sensorial attributes

Since sensory and health-related attributes are perceived by consumers as the most important determinants when choosing fruit and vegetables [38,41], this sub-section presents a brief summary of the influence that these attributes have on the choices of healthy food.

There has recently been a growing interest in the naturalness of products and in a higher health content of food products [42,43]. The healthiness of certain foods may be also determined by their contained phytochemicals and natural compounds, which can have an effect on their sensory characteristics. As a result, these dissimilar levels of compound concentrations, which have a high health function, influence the senses in different ways, with distinct consequences on the sensory appeal of food.

The relevant role of the visual component in the appreciation of food, apart from those of taste and odour, has been widely demonstrated in different sensory studies [44,45], thus evidencing their role in influencing consumers' choices [46,47]. The effects of taste on consumers’ preferences for different agro-food products have been deeply analysed [[48], [49], [50]] and a direct relationship between sensory attributes, such as bitterness/spiciness, and healthiness has been found [51,52]. However, these healthy attributes, that can be poorly appreciated by consumers, can also have a direct effect on colour and taste since they are derived from polyphenols content [53]. Moreover, it has been found that consumers with a high nutritional awareness are more inclined to pay an additional price for food with a high polyphenol content [4,54].

Additionally, although a certain amount of attention has also been paid to the role of smell in the choice of healthy food, and despite having a certain effect on food choices [55,56], it does not appear to be a noteworthy driver of purchase decisions [50]. On the other hand, the effect of colour on the choices of healthy food has been the subject of scant attention by academicians, in particular as an attribute that is able to enhance the consumers’ utility and consequently result in a positive willingness to pay (WTP).

H5 Building upon prior literature and acknowledging the limited research on this topic, the present study aims to investigate the potential correlation between consumers' knowledge and their willingness to pay (WTP) for various colours.

### 2.4 Objectives and research questions

As emphasized in the research background, there is a limited and fragmented understanding of consumers' behaviour concerning the specific sustainable attributes and the influence of colour on their subjective perception of healthy food, particularly regarding vegetables. Consequently, this paper is grounded in the hypothesis that consumers perceive various colour attributes and production methods of food from a health-oriented perspective. The overall objective of this paper has been to assess the effect of different colours on the choice of peppers, according to a hierarchical value of WTP for each visual and sustainable attribute. Considering the medical research demonstrating the direct correlation between colour and varying levels of antioxidants, it became necessary to conduct a comprehensive analysis of how consumers perceive, and hierarchically associate different attributes related to colour and production methods with healthier choices, taking into account their knowledge. Furthermore, as different attributes can elicit distinct levels of preference, this study endeavours to offer novel insights into the influence of colours on willingness to pay, in conjunction with other food characteristics such as interest in antioxidants and consumer knowledge. In addition, we also estimated the WTP for different production methods. Nevertheless, the most detailed and exhaustive analyses were conducted regarding the innovative aspect of colours, which served as the primary focus of this research. Therefore, the paper aims to address the following five research questions in line with the overall objective:

1. Do different colours yield distinct utilities for consumers in their selection of healthier food, resulting in different WTP?
2. Does an interest in antioxidants positively influence the WTP for various colours?
3. Do the socio-demographic characteristics of consumers influence their willingness to pay for colour?
4. Do different production methods have varying effects on utility and WTP?
5. Is there a correlation between consumers' knowledge and their WTP for different colours?

## 3 Methodology

### 3.1 Data collection

Data were gathered using a multi-section questionnaire consisting of four sections: general characteristics of the consumption of sweet peppers; choice experiment; subjective and objective knowledge of the antioxidant properties of peppers; and the socio-demographic characteristics of the participants. The survey was developed using google form, which allowed the survey to be filled in via web.

Different aspects of pepper consumption were investigated in the first section, such as place of purchase, frequency of consumption, importance of seasonality and importance of the characteristics of the peppers. These aspects were investigated using both binary questions (yes or no answers) and Likert scales from 1 to 7, where 1 = not important and 7 = very important, or 1 = never and 7 = very often.

The attributes of the choice experiment were selected after consulting the literature on subjective perception of healthiness and after observing a thematic gap related to the role of colour in food choices, as well as the relationship with conventional, organic and IPM crop growing methods. In fact, there seems to be a gap in the literature on WTP for different colours in healthy foods, while only a few papers have, up to now, compared organic certification with IPM (Ricci et al., 2018; Di Vita et al., 2021). The levels of the price attribute were chosen after conducting a survey to detect the average market price and its variability. Several supermarkets were visited, and the average price per kilogram was obtained and used as the central level of the price attribute. The standard deviation was used to select the maximum and minimum prices to propose to consumers in the choice experiment.

Once the attribute and levels had been chosen and defined for the choice experiment, the issue of generating an efficient design arose [2,36]. To deal with this problem, an optimal D-efficiency design was adopted [57]. This experimental design utilized the covariance matrix derived from the conditional logit estimates obtained in the pre-test phase. Attributes with their levels are indicated in Table 1.

**Table 1:** The attributes and attribute levels adopted in the experimental design.

Attribute	Attribute level
Price	2.29 €/kg; 2.99 €/kg; 3.69 €/kg
Colour	White; Green; Yellow; Orange; Red
Production Method	Conventional; IPM; Organic

**Fig. 1:**
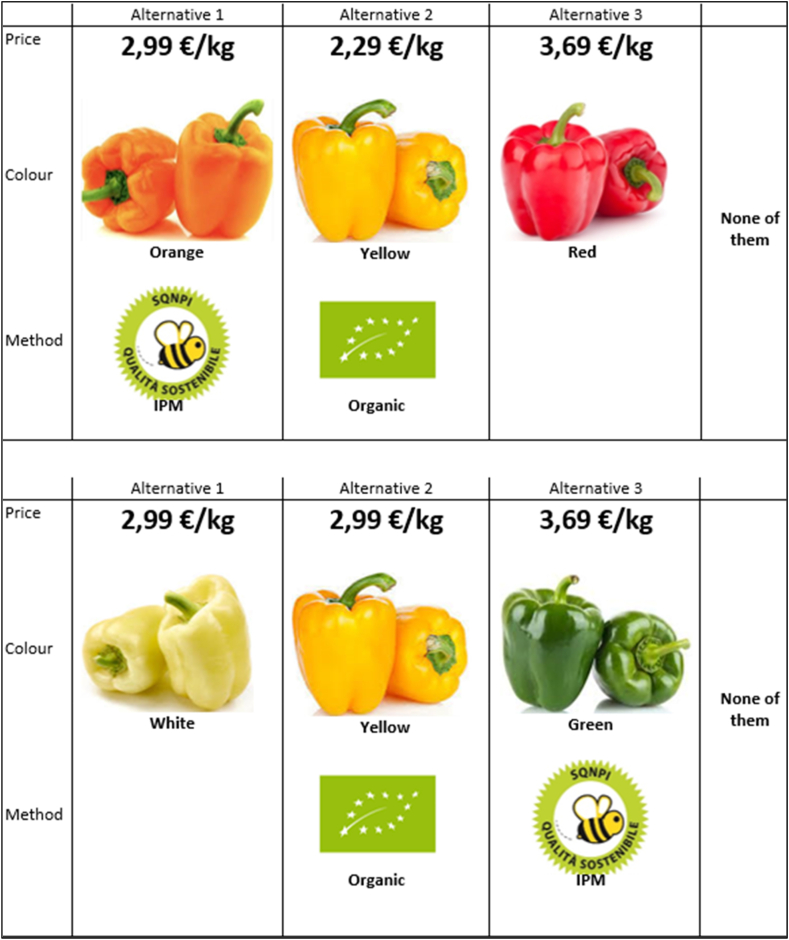
An example of the choice tasks.

Starting from a full factorial design, which was generated from the combination of the attributes and attribute levels, the number of alternatives and tasks that had to be presented to the respondents was reduced by applying a modified Fedorov algorithm to generate a D-efficient design. This method allows the correlations in the data to be minimised in order to estimate coefficients with the lowest possible standard errors [58]. The choice experiment included 12 choice sets divided into 3 blocks. Each set contained three alternatives and the status quo, as indicated in Fig. 1. The participants indicated what product they would choose each time. Whenever the alternatives did not include their preferences, the respondents could select the opt-out or status quo option "none of the presented alternatives" and could then move on to the next choice. Specific information related to the elaboration of the choice experiment is provided in the specific sub section (3.2).

The subjective and objective knowledge of the consumers, related to the quality and properties of peppers, was tested by adapting the constructs developed by Pienak et al. [59], who used them as determinants of organic food consumption. Subjective knowledge was investigated using three items based on a 7-point Likert scale, while objective knowledge was based on a four-item scale, structured with binary questions. Subjective knowledge was evaluated as the average value of the items, while objective knowledge was obtained by assigning a point for each correct answer and then summing the scores obtained by the respondents. Subjective knowledge was evaluated according to Pienak et al. [59], using three items. The following sentences were adopted:

i. “*I know how to recognise the healthy and antioxidant-derived properties of vegetables, in particular for sweet peppers”;*
ii. “I am able to associate the antioxidant properties with the colour of sweet peppers”.
iii. *“People who know me believe I am competent in recognising the antioxidant qualities of peppers”.*

Four items were used for objective knowledge, with yes or no answers, as follows:

i. “*Yellow peppers contain more antioxidants than red peppers”.*
ii. “Cooking peppers allows all the naturally present compounds (e.g., vitamins, antioxidants) to remain intact”.
iii. “Vitamins are scarcely present in peppers”.
iv. “The amount of antioxidants in peppers can be affected by the colour of the skin”.

Consumers’ knowledge was assessed without providing them information or clues related to the characteristics or properties of peppers. Thus, the results related to knowledge can be considered as consumer knowledge without information.

Data was collected in 2021, from April to August, using a snowball sampling method that involved sharing a link on different social networks [60,61]. Snowball sampling is also known as “chain referencing” and can be considered as a non-discriminatory and non-probabilistic method that allows observations to be exponentially collected while overcoming the difficulty of reaching a dispersed population [60]. Moreover, this sampling method permits data to be collected through social networks, since a questionnaire can be shared with other respondents [62]. The link was shared online in Italy on social networks that deal with aspects related to food, horticulture and the consumption of healthy food. The introductory part of the survey contained important indications on how to correctly fill in the questionnaire in order to obtain reliable answers. In this respect respondents were provided with additional information about what IPM was. In addition, specific details were also provided about the role of antioxidant as cue for healthy properties, highlighting that a certain association between colour and antioxidant content exists.

The respondents were informed that the survey was targeted to consumers over 18 who were actually responsible for purchasing food. Moreover, the research was carried out in line with the ethical standards of the Declaration of Helsinki. Participants were informed about the content and purpose of the study, and all of them participated voluntarily and gave their informed consent.

The data gathering process allowed a sample to be collected of 1053 consumers. The sample data has been weighted based on gender and age^1^ to better reflect the demographics of the Italian population. This weighting process helps to account for any potential imbalances, ensuring that the findings of the study are more representative of the broader population [63]. Consumers answered the choice tasks correctly and were therefore considered for the WTP estimates. Subsequently, a screening was performed on the consumers who had completed the socio-demographic section, and 997 complete records, presented in Table 2, were thus obtained and were then used to perform Seemingly Unrelated Regression (SUR). Table 2 also indicates the codification of the variables used in SUR regression. The constructs on socio demographics, interest in antioxidant content and knowledge were selected to deal with the research questions, because they are considered important drivers of consumers’ choices [64].

**Table 2:** Socio demographic characteristics of the sample no. = 997.

Variable	Category	Coded	Sample data (%)	National Data (%)
Gender	Male	0	48.19	48.19
	Female	1	51.81	51.81
Family members	1-2 members	1	51.02	60.90
	3-4 members	2	45.03	34.10
	More than 4 members	3	3.94	5.00
Age Cohort	Millennials	1	28.10	28.10
	Generation X	2	36.56	36.56
	Baby boomers	3	21.30	21.30
	Older Generations	4	14.04	14.04
Education	Up to middle School	1	6.17	36.52
	High School	2	41.54	49.59
	University	3	40.01	13.35
	Higher education	4	12.28	0.54
Interest in antioxidants	No	0	26.95	N.A
	Yes	1	73.05	N.A
Subjective knowledge	Mean (Standard Deviation)	From 1 to 7 From 0 to 4	3.38 (1.66)	N.A
Objective knowledge	Mean (Standard Deviation)		2.98 (0.88)	N.A

N.A. = Not Available.

### 3.2 Data Analysis^1^

#### 3.2.1 Choice model

The choice experiment was adopted as a stated preference elicitation method as it is useful to simulate a real choice [65] and to investigate the preferences of consumers for the attributes of peppers, and in particular for their colours. The model lies on the Random Utility Theory [66], in which consumers’ choices are affected by the probability of the utility of a particular combination of attributes, as derived from the sum of the characteristics of a product, is higher than the utility generated by the other alternatives in the same choice set.

On the basis of this consideration, the utility (U) perceived by consumers for a specific configuration, *s*, is a function of a deterministic part (V) and a stochastic element, *e,* as indicated in equation (1).

(1) $$U_{is}=V_{i}\;\left(X_{s}\right)+e_{is}=\beta_{i}X_{s}+e_{is}$$

In equation (1), $X_{s}$ is the vector of the detectable characteristics of the products, *s*, and $\beta_{i}$ represent the vectors of the parameters that quantify the direction and magnitude of the association between the consumers' characteristics and the attribute levels of the products. $\beta_{i}X_{s}$ were obtained by considering attribute levels as a set of dummy variables allowing the estimation of coefficients for each level. Conventional production and white colours were considered the base level for the comparison of coefficients to avoid the dummy trap.

When the rationality of individuals is assumed, the choice of the *I* respondents occurs when the *s* alternative, compared with the other *l* options in the choice set, provides a higher utility ($U_{is}$) than the other evaluated products ($U_{il}$), as indicated in equation (2).

(2) $$U_{is}>\;U_{il}\;\forall \neq s$$

Utility and WTP assessments were conducted using a mixed logit conditional model and utilising a maximum likelihood estimator (Altobelli et al., 2021). In this context, the $\beta_{i}$ parameters were considered random and to follow a normal distribution $N∼\left(\mu ,\sigma^{2}\right)$ over the sample. The advantage of random parameters is related to the fact that individual coefficients can be obtained allowing the analysis of distribution for $\beta_{i}$ parameters to be conducted and capturing unexplained heterogeneity of individual preferences related to attributes [67].

#### 3.2.2 SUR regression

A Seemingly Unrelated Regression model was considered to describe the relationships between the WTP estimates obtained from the choice model and the different attribute levels linked to colours. This method is used when a particular phenomenon can be conditioned by certain correlated preferences, such as in the case of the WTP for a particular colour, where the choice can be related to the preference of an individual for certain colours. According to this consideration, the relationships detected by a linear model might not describe the dependent variable accurately. In this case, a system of equations is required. Results provided by multivariate SUR model can be more reliable compared to those estimated by subsequential OLS in terms of statistical efficiency [68]. In fact, SUR regression can be considered as a multivariate linear regression tool that consists of four related linear equations [69], as described in equation (3).

(3) $$\left\{ \begin{array}{c} {WTP}_{green\;colour,i}=x^{\prime }\beta_{green+}{\;e}_{green,i}\\ {WTP}_{yellow\;colour,i}=x^{\prime }\beta_{yellow+}{\;e}_{yellow,i}\\ {WTP}_{orange\;colour,i}=x^{\prime }\beta_{orange+}{\;e}_{orange,i}\\ {WTP}_{red\;colour,i}=x^{\prime }\beta_{red+}{\;e}_{red,i\;} \end{array}\right.$$

In the here presented system of equations, x represents the vector of the covariates used to predict WTP for different colours, while *e* is the related error term.

Generation of choice set and future analysis involving mixed logit regression and SUR were conducted using Stata® 16.

## 4 Results

The random coefficients obtained from the mixed logit regression, as presented in Table 3. In economic terms, the coefficient estimates are associated with the marginal utility ascribable to the related attributes. All the coefficients, except for price, are considered as random and vary significantly across consumers, thus the assumption of fixed parameters is avoided. Finally, the estimated coefficients were obtained by excluding the white colour and the conventional cultivation method. Consequently, the utility and the related WTP were estimated by referring to the values of white peppers and to the conventional production method. The qualitative importance and magnitude, as well as the direction of the effects of the attribute levels allowed a preliminary interpretation of the model to be made.

**Table 3:** Mixed logit results and WTP for the attribute levels.

Attribute	Coefficient	Robust std. err	t-stat	*p*-value	Marginal WTP
Price	−1.569	0.150	−10.46	<0.001	
Green Colour	0.882	0.297	2.97	0.003	**0.573**
Yellow Colour	2.888	0.340	8.51	<0.001	**1.891**
Orange Colour	2.078	0.339	6.13	<0.001	**1.378**
Red Colour	3.565	0.290	12.27	<0.001	**2.352**
IPM	1.674	0.318	5.26	<0.001	**1.060**
Organic	2.121	0.301	7.06	<0.001	**1.377**
Opt-out	−3.560	0.392	−9.08		
σ (Green C.)	1.906	0.359	5.32		
σ (Yellow C.)	2.251	0.428	5.26		
σ (Orange C.)	1.691	0.259	6.53		
σ (Red C.)	2.283	0.440	5.18		
σ (IPM)	2.351	0.401	5.86		
σ (Organic)	2.599	0.515	5.04		

Each of the attribute levels related to the colours has a positive effect, compared to the white colour. The red colour provides the highest utility to consumers (3.565), and this is followed by the yellow colour (2.888), while the green colour is the least important for consumers, in terms of utility estimate (0.882). Since each colour shows a positive coefficient, the white peppers were used as the basis for the estimates, as it was considered the least attractive for consumers throughout the analyses.

When considering the production method, IPM and organic labels can both be considered as attributes of a certain interest for consumers, compared with the conventional method of production. The organic method is considered more important than IPM production, since the presence of this label has the largest coefficient (2.121). It is interesting to observe that the orange, yellow and red colours are deemed to be more important than the production methods, thus suggesting that colour is an intrinsic characteristic that can generate a higher utility than certain extrinsic attributes linked to sustainability, such as the production method. Among the different colours, only the green one provides less utility than IPM and the organic method of production.

The WTP for each respondent can be calculated from the estimated parameters as the marginal value of the attribute in monetary terms. This is done by computing the ratio of the parameter estimated for the non-monetary attribute to the price parameter, and then multiplying the result by negative one [70]. The values of WTP are related to the utility coefficients, follow the same direction and have a similar importance, but they also provide a concrete way of assessing the magnitude of the estimates. In fact, the red colour is the most valued attribute, and has obtained a WTP of 2.352 €. Like the rest of the colours, yellow obtained a WTP of 1.891 € while the estimated WTP for the orange colour was 1.378 €. Finally, the green colour was the least valued by consumers; in fact, the mean estimated WTP was 0.573 €.

As far as the cultivation method is concerned, the presence of the organic attribute generated a mean WTP of 1.377 € while the presence of IPM certification obtained a mean WTP score of 1.060 €. These results suggest that colour is capable of generating a higher WTP for consumers, thus indicating the importance of this attribute. However, the production method can also enhance the value of productions, and organic certification seems to be more effective in producing a higher WTP than crops grown under IPM.

**Fig. 2:**
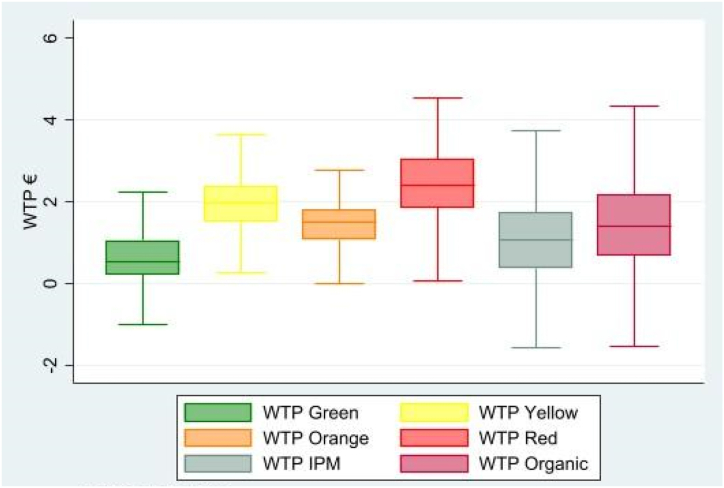
Distribution of the WTP for the different product attributes.

These results are tabulated in Table 3, where they can be used to achieve a brief interpretation; however, the distribution of WTP over the respondents is explained more clearly in Fig. 2, where box plots are provided for both the colours and the production methods. In fact, WTP values are obtained for each consumer,^2^ thus allowing a graphical representation of them to be made and further analysis to be conducted to explain what pushes consumers WTP towards different types of peppers.

Following Campbell [71] and Weituschat [70], the WTP values were analysed using a Seemingly Unrelated Regression model that represents a multivariate linear regression, which in turn allows the variation in WTP to be estimated, and to account for a possible correlation of the error terms across the equations.^3^ Using this model, which is reported in Table 4, the coefficients can be interpreted as marginal effects derived from the independent variables and, in economic terms, as the variation in euros.

**Table 4:** SUR regression results obtained using WTP for colours as dependent variables.

Regressor	Green colour	Yellow colour	Orange colour	Red colour
Antioxidant interest	0.004	0.018	0.039***	0.074***
Age Cohort	−0.005	−0.043***	−0.020***	−0.050***
Gender	−0.001	0.043***	0.037***	0.038**
Family members	−0.011	−0.011	−0.010	0.031**
Education	0.064***	0.051***	0.034***	−0.001
Subjective knowledge	0.011**	−0.007*	−0.008***	−0.017***
Objective knowledge	−0.015***	−0.002	−0.001	0.022***

Note: Asterisks indicate statistically significant variables at: *p ≤ 0.1; **p ≤ 0.05; ***p ≤ 0.01; Breusch–Pagan test of independence: χ2(6) = 43218.4, *p*-value <0.0001.

The covariate for the effect of consumers’ interest in the presence of antioxidant compounds is significant for the red and orange colours. It is interesting to note that the marginal effect is higher for the red pepper typology, that is, where antioxidant compounds are more concentrated, thus suggesting that a relationship exists between interest and colour that could be considered a proxy for antioxidant content. This information indicates that consumers are more willing to pay for red peppers and, albeit to a lesser extent, for orange peppers, whenever they are also interested in the antioxidant content.

A significant effect of the age cohort is observed for the yellow, orange and red colours. Considering the negative sign, it can be observed that as the age increases, the number of consumers who are willing to pay for such colours decreases. The observed trend suggests that peppers are consumed more by young people who are more interested in and willing to pay for such products.

The information about gender is also significant for the yellow, orange and red colours. The positive sign indicates that women are more willing to pay for these peppers than men. However, this trend is not observed in the case of large families, and those families with more components are in fact only more willing to pay for red peppers, while no significant effects are observed for the other colours.

Significant WTP values have been observed for education for the green, yellow and orange colours. The observed positive coefficients indicate that as the education level increases, the WTP for these products increases, and it has been observed that consumers are willing to pay more for green peppers.

Finally, subjective knowledge and objective knowledge show rather surprising trends. In fact, although objective knowledge is not significant for yellow and orange peppers, a significant WTP is observed for the green and red colours. Since these products are those with lower and higher antioxidant contents, respectively, this result could appear slightly controversial, and it requires further explanation. In this case, the sign is particularly important to understand the role of knowledge and the relationship with the antioxidant content.

Subjective knowledge positively affects the WTP for the green colour, thus indicating that consumers who believe they are aware of the properties of peppers are more willing to pay for this colour. Conversely, since the effect of objective knowledge is negative, consumers who are in fact aware of the properties of peppers are less willing to pay for green peppers.

The results related to the red colour are opposite. In fact, subjective knowledge negatively affects the WTP for the red colour, thus suggesting that consumers who believe they are aware of the properties of peppers are less willing to pay for this product. Since objective knowledge is positively related to the WTP for red peppers, consumers who possess a higher level of knowledge of the properties of peppers are more willing to pay for the red colour.

The negative effect of subjective knowledge is also significant for yellow and orange sweet peppers, thus suggesting that, as the polyphenol content of a product increases, WTP decreases in relation to the level of the consumers' subjective knowledge. These results suggest that there is a relationship between consumers’ knowledge and the willingness to pay for colours and, since the antioxidant properties are closely correlated to each other, this occurs in particular for red peppers.

## 5 Discussion

The primary objective of this study has been to offer fresh insights into consumers' preferences for food products characterized by distinct visual and sustainable attributes, which can be reasonably associated with healthiness due to antioxidant content. The study highlights the pivotal elements influencing consumers during the purchasing phase, with a particular focus on the assessment of the impact of various health-related colours on consumer perception and choices. Employing five different chromatic scales based on differently coloured sweet peppers, as per the classification by Matsufuji et al. (2007), the subsequent section organizes and discusses the study's findings in response to the research questions (RQs).

The obtained results corroborate the hypothesis that hierarchy preferences exist between different levels of attributes related to colour and production method. In particular, it seems to be well-defined in favour of warm colours. In fact, the intrinsic cues linked to the colours of products with a high polyphenol content, that is, red, yellow and orange, were deemed to be more important than the extrinsic characteristics of the product. This preliminary result is in line with previous findings in the literature, whereby consumers’ preferences are mainly influenced by intrinsic and sensory characteristics in vegetable purchasing decisions [72]. Similarly, another study found that the effects of extrinsic characteristics are generally less noticeable than intrinsic attributes, even for processed vegetables [73]. Accordingly, the results of the present study indicate that consumers seem to value sensory attributes more, as in the case of warm colours, than the extrinsic characteristics linked to certified sustainable methods of production, such as organic and integrated practices.

This finding makes it possible to assert that consumers consider the role of warm colours as being of primary importance. Conversely, the role of white and green colours shows a completely opposite trend. In fact, these two colours are not considered important for the choices of consumers. This latter result reflects the results of a recent study on the purchase intention towards ready-to-eat salad, in which the appreciation of colour was analysed [73]. As in our case study, consumers evaluated the green colour as being of scarce importance, although they appreciated salads characterised by manifold colours, such as orange and purple, much more. This is probably because these colours arouse emotions of health and wellness [73].

Summarising this result, it has emerged that visual cues influence consumers’ choices of colour in different ways and the effects of extrinsic cues are generally less prominent.

**RQ 1.** Concerning the first research question, we can positively respond to it. In fact, our study has revealed that the initial assumption, that is, that different colours provide consumers with different utilities in the choices of healthy foods, was correct. Nevertheless, this utility progressively decreases according to the following order: red-yellow-orange-green. Conversely, the white colour has revealed a negative utility. Therefore, the red colour is the preferred colour of the sampled consumers. In addition, although unknowingly, the consumers chose the colour most likely to guarantee a higher health content. In fact, red sweet peppers ensure the most antioxidant activity and provide more improved quality and beneficial effects for the maintenance of health than sweet peppers of other colours [24].

Consequently, the results are partially in line with other studies, although they do not appear completely consistent. In fact, quite controversial results on consumers' utilities derived from the choice of the red colour appear in the literature. A recent study on consumers’ perception of different coloured food labels has highlighted that red is often associated with a risk or danger and failure, and consequently it could lead to a decision to not choose it [74]. Moreover, another study indicated that consumers believe fresh potatoes are healthy foods and mainly expressed their preferences for white and purple skin. However, red was not considered relevant. A predominance of yellow over the red colour has instead been observed [75].

Another study, based on consumers’ preferences for edible flowers, revealed that red and orange were the favourite colours for the sampled respondents [76]. Therefore, the findings of our study confirm the results of a previous research on the existence of specific market segments for warm colours, such as orange, red and yellow [25], since the sampled consumers indicated a specific interest in diversified colours, albeit different from green. Furthermore, it is possible that colour preferences may depend on the type of product, thus generating different utilities.

The different utilities perceived by consumers lead to statistically significant and different WTP. In fact, taking a look at the mixed logit results, it emerges that respondents express a positive WTP for all the attributes, except for price. In the case of the WTP for colours, our research has first confirmed the belief that colour positively affects consumers’ willingness to pay, as pointed out by Yin et al. [77]. In other words, red peppers show the highest WTP values. This result confirms some results in the literature, despite the existence of a certain heterogeneity in colour values of fresh food. Colour has already been explored in depth as far as the logo, label and packaging of food products are concerned, but the research that has analysed its role in depth in such fresh produce as fruit and vegetable is rather scarce. However, one study, where consumers declared a higher WTP for red skin tones, has shown that the WTP values for peaches are also positive [78].

**RQ 2**. Interesting insights have also emerged for the role of antioxidants in consumers' choices. Taking into account the SUR regression results, which are based on different consumers’ WTPs for each colour, it emerges that the red colour, and to a lesser extent, orange peppers, have a positive and significant correlation with an interest in antioxidants. This makes it possible to establish that the consumers with the highest interest in antioxidants are those who show the highest WTP for colours related more to an antioxidant content, and red peppers in particular, followed by the orange type, while the role of yellow is less significant. Yellow is in fact a colour that consumers associate with a normal state of health in their preference for food [79]. However, no significant relationship was found between antioxidant awareness and the green and yellow colours.

Our study, albeit indirectly, confirms the current trend of consumers, that is, to be more willing to pay for food that is high in both natural and enriched antioxidants [[80], [81], [82]]. In addition, our findings are partially consistent with those of an economic experiment based on the influence of visual attributes of food packaging. Again, in that case, the red colour was preferred to yellow, while the highest subjective perception of healthy product was for red and yellow packaging [83].

In light of these premises, the existence of a hierarchical correlation can be deduced between the interest in the antioxidant properties of peppers and distinct colours, due to the presence of different antioxidant contents. This implies that the consumers who are looking for healthier sweet peppers tend to choose, albeit randomly, those that are characterised by warm colours which, by chance, are also those that have the highest antioxidant content.

**RQ 3** As regards the effect of socio-demographic characteristics, our study highlights the roles of gender, age, education, and number of family components in affecting the willingness to pay for colour*.* Therefore, these outcomes have allowed us to positively respond to the fourth research question. Again, in this case, the key role of the red colour emerges for large families, females, and young consumers.

As regard age, younger consumers show a high propensity for red, orange and yellow. This result is quite consistent with those of previous studies. In fact, the younger generation is more motivated by coloured signs, although it is less reactive to healthy information [84]. In addition, our result is also partially consistent with other studies that have highlighting how youth, happiness and energy are conveyed by the red colour, as is the case of some highly reputed and appreciated food products, such as coca cola and ketchup, among the younger generation [85].

Our study has also revealed that red is also relevant for large families, and that red, orange and yellow peppers are particularly important for women. The results pertaining to women are in line with previous research that focused on the role of colour in influencing the buying behaviour of female consumers [86,87]. Generally, women are positively influenced by orange, yellow and green, but they are particularly attracted by red goods, which appear to provide a higher level of satisfaction [85]. Therefore, our study confirms that, also for fresh vegetables, a different attitude towards colours exists between gender, since women react positively when choosing coloured peppers.

As for the size of a family, the outcomes of our study show that households with a large number of components react differently to colour. This result appears to be in line with early empirical evidence that discovered how the size of a household influences the buying process of sweet pepper consumers. Household size, in fact, seems to characterise a specific segment of the market [25], and in addition a keen interest in small sized peppers in large families has been observed [88]. Some of these studies focused on the behaviour and attitudes of the consumers of sweet peppers, but no survey has provided empirical evidence on the association between this socio-demographic characteristic and colour preferences.

Finally, a direct correlation has been found between education and colours. More educated consumers primarily prefer green and yellow colours and, albeit to a lesser extent, the orange one. This outcome confirms previous research on clothing and furniture consumers and highlights the relationship that exists between colour preferences and educational background [89]. The preference for green pepper by more educated consumers can be traced back to tradition. Probably this group of people associates vegetables with prototypical colours, such as red for tomatoes or orange for carrots, and when these deviate from their typical colour they may receive comparatively lower attractiveness ratings [90]. Green colour is widely recognized as the most common and typical hue for sweet peppers, making it highly familiar and widespread. However, there is a lack of studies on the correlation between education and colour preferences in food. One of the few studies that exist has evidenced how maize consumers with higher education levels choose white over yellow attributes [91].

**RQ 4.** The fourth research question aimed to assess the impact of different production methods on generating utilities and consumers' willingness to pay. Indeed, gauging the value consumers place on pesticide reduction is another crucial aspect of evaluating the potential market for farmers looking to capitalize on the growing demand for pesticide-free products [92].

In this regard, we assessed the potential willingness to pay (WTP) for organic and Integrated Pest Management (IPM) sweet peppers. Our research findings align with the growing preference for organic products, which can be attributed to their well-established certification standards. Notably, organic products have received positive feedback from consumers, as evidenced by studies such as Li and Kallas [93]. Additionally, Pascale et al. [94] conducted a comprehensive study across four European countries, investigating consumers' willingness to pay (WTP) for apples associated with various pesticide-use certifications. The results consistently revealed that European consumers exhibited a strong inclination towards apples certified for reduced pesticide use, with a particular preference for organic production methods.

Concerning the willingness to pay (WTP) for the cultivation of peppers, the sustainability production method holds greater significance and garnered the interest of the respondents. Organic certification yielded a higher WTP compared to peppers grown under an Integrated Pest Management (IPM) approach. This outcome aligns closely with prior research emphasizing the importance of an organic label and IPM [36], where consumers demonstrated a moderately positive inclination to pay for agro-food products produced using both organic and integrated pest management practices [36].

Although, in some cases, it has been found that an organic label barely contributes to increasing the utility of sweet pepper consumers [95], a positive willingness to pay for organic vegetables and peppers is well-established, as has been confirmed in several studies [96].

**RQ5.** As regard the last research question, we have found a certain relationship exists between consumers' knowledge and their preference for certain pepper colours with different antioxidant contents. The findings related to objective and subjective knowledge are particularly interesting. The results can be summarised in the following sentence: *“Those who really have knowledge about pepper nutrition properties pay more for red pepper, and those who do not know about them pay more for green peppers”.* Such a result sets limits on the role of subjective knowledge, as regard the potential consumers’ perception about the real healthiness of a product. Thus, this implies that, although nutrition knowledge is deemed to be a likely forecaster of healthiness [97,98], objective knowledge could be considered a good predictor of food products rich in antioxidant and then a probable relation with subjective perception of healthy food could exist. Therefore, our findings highlight the limits of subjective knowledge in the conscious healthy food choice phase. This confirms the results of a study on functional foods in which high subjective knowledge was found to lead to a decrease in the adoption of such food in the diet [99].

Lastly, it can reasonably be mentioned that informed consumers show a greater aptitude and propensity towards potential healthy food, as proven in recent studies on the role that individual and objective knowledge plays in the perception of different health attributes [100,101].

## 6 Conclusion

### 6.1 Main results

This study explores consumers' perceptions of healthier peppers by examining their preferences for color and estimating the willingness to pay (WTP) for various colours and production methods. It associates these preferences with diverse consumer characteristics, including socio-demographic features and antioxidant interest. The findings reveal hierarchy preferences for warm colours and establish a correlation between consumers' interest in antioxidants and their WTP for specific colours. Furthermore, the study underscores the significant impact of socio-demographic factors and production methods on influencing both color preferences and WTP. It emphasizes objective knowledge as a pivotal factor shaping consumers' WTP.

More in detail, consumers consider the role of warm colours**,** such as red, yellow, and orange, as being more relevant than sustainable labels, for example, organic and IPM. Red has been found to be the prominent colour in terms of utility, WTP and overall subjective perception of healthier food and it is followed by yellow and orange. Green is also considered as an important colour attribute for more educated consumers, probably because of its wide diffusion and familiarity. Overall, consumers exhibit hierarchy preferences for warm colours, emphasizing intrinsic cues over extrinsic characteristics. In addition, we have found, through the WTP estimates, that the red colour, followed by yellow and orange, is considered by consumers to be the most capable of ensuring a higher health content.

Furthermore, the findings revealed that consumers with a strong interest in antioxidants express the highest willingness to pay (WTP) for colours associated with higher antioxidant content. This suggests that consumers seeking healthier sweet peppers tend to prefer warm colours with increased antioxidant levels. The observed connection between consumers' interest in antioxidants and their WTP for specific colours suggests a correlation between color preferences and perceived health benefits.

According to our outcomes, the sociodemographic characteristics of the consumers certainly play a role in predicting the consumers’ WTP for different colours. Female, younger consumers and large families are the consumers who are most interested in the red colour, and thus those who associate a higher subjective perception of healthier peppers with this colour.

However, the **s**tudy contributes to the growing preference for organic products, with consumers exhibiting a higher WTP for organic certification compared to IPM, aligning with existing research on the positive perception of organic products.

Finally, concerning knowledge constructs, it is evident that objective knowledge is more closely associated with a heightened awareness of the health properties of peppers compared to subjective knowledge. This connection results in a positive willingness to pay (WTP) for the red color, emphasizing the limitations of subjective knowledge in the conscious decision-making process for healthy food choices.

### 6.2 Novelty of the study

This study introduces several noteworthy contributions. Specifically, the research delves into consumers' subjective perception of healthier peppers, emphasizing their connection to colour. The study estimates the willingness to pay (WTP) for different colours while associating these preferences with various consumer characteristics. Notably, colour is employed as a cue for inferences about credence attributes such as healthiness.

In terms of sociodemographic factors, the conducted survey provides novel empirical evidence on the association between these factors and colour preferences by simultaneously evidencing the role of gender, household size and education.

Furthermore, this paper breaks new ground by exploring the interplay between colours and WTP in conjunction with other food characteristics, such as production methods, providing valuable hierarchical insights into consumer preferences.

A significant finding is the positive correlation discovered between interest in antioxidants and colours. This implies that consumers with a keen interest in healthy food properties are inclined toward specific sensory choices, as reflected in their colour preferences.

Lastly, the study reveals a diverging role between subjective and objective knowledge when it comes to health-conscious choices, adding a nuanced layer to our understanding of consumer decision-making in the realm of healthy food preferences.

### 6.3 Theoretical, marketing and policy implications

The results of this study could pave the way towards several theoretical, marketing and policy implications.

Starting from the theoretical aspects, this survey provides preliminary insights into consumers' Willingness to Pay (WTP) for different colours of peppers, establishing a hierarchy among them. This contributes to the theoretical understanding of consumer preferences and choices related to visual attributes in the context of healthier food. Moreover, this paper consolidates the results available in literature concerning the importance of IPM for product differentiation. Additionally, particular and novel insights have emerged for the different roles played by subjective and objective knowledge as WTP predictors.

This paper also provides useful marketing implications, in terms of consumers’ motivations and subjective perception of healthier food. Color is identified as a motivational variable in healthier food products. Marketers can leverage this information to tailor their marketing strategies, emphasizing the visual appeal of peppers to align with consumer preferences.

Furthermore, the findings in this paper could be utilized by producers for the thoughtful selection of pepper cultivars, addressing consumers' preferences and enhancing satisfaction. Producers may also gain advantages by choosing pepper cultivars based on color preferences, thereby improving their ability to meet consumer demand effectively. The study highlights the importance of Integrated Pest Management (IPM) for product differentiation. Farmers and producers can consider adopting organic or IPM growing methods to enhance the value of their pepper productions. This information is crucial for those seeking to distinguish their products in the market based on sustainable and environmentally friendly practices.

As for policy implications, fresh vegetable producers and the industry in general, as well as policy makers could plan a campaign to inform citizens about the different health benefits of distinct colours of fresh food produce, as in the case of fruit and vegetables.

Finally, this paper provides useful insights for institutions and producers, as it presents novel information related to sustainable production methods and to the effectiveness of organic and IPM certification in generating a higher consumers’ WTP.

### 6.4 Limitations and further research

The study is affected by some limitations that should be considered when generalising the results. The snowball sampling method suffers from some limitations, as it represents a non-probabilistic method. This approach may introduce biases such as selection bias and non-representativeness, potentially affecting the diversity and representativeness of the sample. While we have attempted to mitigate these issues by weighting the sample against national statistics on gender and age, this does not fully address other potential biases. Moreover, the choice experiment is affected by a hypothetical bias that reduces the reliability of the estimates. Finally, only three attributes were evaluated to obtain a robust design, and the choice of peppers may depend on several attributes, consequently the results could vary if new combinations of attributes and levels were to be considered.

As far as future research is concerned, it would be desirable to conduct an in-depth organoleptic analysis of consumers' preferences and to include, if possible, other sensory attributes, such as taste and smell, and/or other colours, for example, purple and brown. Finally, a non-hypothetical approach, such as an experimental auction, could be taken into consideration, as this would probably be more effective in obtaining more reliable estimates. Furthermore, if a multi attribute evaluation approach were to be adopted, other attributes and predictors could be chosen and tested to assess the perceived healthiness of products in terms of consumers’ preferences and WTP. Finally, the role of psychometric variables can be assessed using validated scales, also related to perceived healthiness to find a direct relation with colours.

## Ethics statement

For this study type, our university does not require approval from an ethics committee; however, obtaining informed consent from participants is mandatory. The studies were conducted in accordance with local legislation and institutional requirements, with participants willingly providing written informed consent to partake in the study.

## CRediT authorship contribution statement

**Giuseppe Di Vita:** Writing – review & editing, Writing – original draft, Supervision, Methodology, Formal analysis, Data curation, Conceptualization. **Raffaele Zanchini:** Writing – review & editing, Writing – original draft, Software, Methodology, Formal analysis, Data curation. **Daniela Spina:** Writing – review & editing, Writing – original draft, Methodology, Formal analysis, Data curation. **Antonella Vastola:** Writing – review & editing, Visualization, Investigation. **Mario D'Amico:** Validation, Supervision, Resources, Project administration, Conceptualization. **Francesco Caracciolo:** Writing – original draft, Supervision, Software, Methodology, Formal analysis, Data curation, Conceptualization.

## Declaration of competing interest

The authors declare that they have no known competing financial interests or personal relationships that could have appeared to influence the work reported in this paper.

## Footnotes

1 The data were sourced from official national statistics, as provided by the Italian National Institute of Statistics (ISTAT). More information can be found on their website: https://www.istat.it/en/population-and-households.

## Footnotes

2 The method proposed by Revelt and Train (2000) has been used to obtain individual-specific parameters from the mixed logit estimates. The Stata command 'mixlbeta' was used in this process.

3 The application of SUR was guided by the premise that while WTP for different color attributes might be driven by different observable factors, there could also be unobserved factors that influence these preferences in a correlated manner. For instance, underlying aesthetic preferences or cultural perceptions that are not directly measured in the study could affect WTP for multiple color attributes simultaneously. This approach has been empirically validated by the results of the Breusch–Pagan test for independent equations.
