# Supplementary material for: Simply red? The effects of distinct colours and sustainable production methods on the consumers’ preferences for healthier sweet peppers
Source: Heliyon. 2024 Apr 12;10(8):e28661. doi: 10.1016/j.heliyon.2024.e28661 (PMC11031798; doi:10.1016/j.heliyon.2024.e28661)
Supplement: Multimedia component 1 [file mmc1.docx]

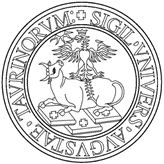


SURVEY ON THE CONSUMPTION OF PEPPERS

Card no. …………

Province: ………….…………………………………………………………………

Municipality: ………………………………………………………………………………

INTRODUCTION TO THE INTERVIEWS

*******************************************************************************

Dear Sir/Madam,

the University of Turin is conducting a survey on the consumption of peppers. We would be grateful if you would agree to answer some questions on this topic. I would like to inform you that the aim of the interview is not to sell products and that any information you should provide will be used in an anonymous and aggregated manner and only for research purposes.

To give validity to the answers, I kindly ask you to fill out all sections and answer IF you are the one doing the shopping.

Thank you in advance for your participation!

Do you authorise the processing of your personal data indicated in the survey in accordance with Legislative Decree no. 196/03 and subsequent amendments, and with European Regulation 679/2016?

1. Yes 
2. No. In that case, thank you just the same for your time 

If you turn out to be a consumer of peppers, this will be followed by a series of questions about your consumption and purchase of such products. However, if you answer NO, the questionnaire concludes here. Thank you for your time regardless.

CHOICE EXPERIMENT

Let’s begin with a brief consumer choice experiment. In the following sections, we ask you to imagine yourself at the supermarket to buy some peppers in 1kg packages. The products vary in the following characteristics:

*(We have attached all the tasks in a separate file)*
